# Intestinal Region-Specific and Layer-Dependent Induction of TNFα in Rats with Streptozotocin-Induced Diabetes and after Insulin Replacement

**DOI:** 10.3390/cells10092410

**Published:** 2021-09-13

**Authors:** Nikolett Bódi, Lalitha Chandrakumar, Afnan al Doghmi, Diána Mezei, Zita Szalai, Bence Pál Barta, János Balázs, Mária Bagyánszki

**Affiliations:** Department of Physiology, Anatomy and Neuroscience, Faculty of Science and Informatics, University of Szeged, Közép fasor 52, H-6726 Szeged, Hungary; lalitha.biochem87@gmail.com (L.C.); afnanaldoghmi1217@gmail.com (A.a.D.); mezei.diana@bio.u-szeged.hu (D.M.); zszalai@bio.u-szeged.hu (Z.S.); bbencep96@gmail.com (B.P.B.); jbalazs@bio.u-szeged.hu (J.B.); bmarcsi@bio.u-szeged.hu (M.B.)

**Keywords:** tumour necrosis factor alpha, cytokine, myenteric neurons, neuronal environment, duodenum, ileum, colon, diabetes, insulin

## Abstract

Tumour necrosis factor alpha (TNFα) is essential in neuroinflammatory modulation. Therefore, the goal of this study is to reveal the effects of chronic hyperglycaemia and insulin treatment on TNFα expression in different gut segments and intestinal wall layers. TNFα expression was mapped by fluorescent immunohistochemistry and quantitative immunogold electron microscopy in myenteric ganglia of duodenum, ileum and colon. Tissue TNFα levels were measured by enzyme-linked immunosorbent assays in muscle/myenteric plexus-containing (MUSCLE-MP) and mucosa/submucosa/submucous plexus-containing (MUC-SUBMUC-SP) homogenates. Increasing density of TNFα-labelling gold particles is observed in myenteric ganglia from proximal to distal segments and TNFα tissue levels are much more elevated in MUSCLE-MP homogenates than in MUC-SUBMUC-SP samples in healthy controls. In the diabetics, the number of TNFα gold labels is significantly increased in the duodenum, decreased in the colon and remained unchanged in the ileal ganglia, while insulin does not prevent these diabetes-related TNFα changes. TNFα tissue concentration is also increased in MUSCLE-MP homogenates of diabetic duodenum, while decreased in MUC-SUBMUC-SP samples of diabetic ileum and colon. These findings support that type 1 diabetes has region-specific and intestinal layer-dependent effects on TNFα expression, contributing to the regional damage of myenteric neurons and their intestinal milieu.

## 1. Introduction

Distinct structure and function as well as genetic and developmental features fundamentally define the appropriate intestinal milieu in the small and large intestine and even in their subregions [1,2], and pathological stimuli differently affect this regional molecular environment. Among others, type 1 diabetes has strictly region-specific effects on the microbial composition [3,4], antioxidant defence or oxidative status [5] of different gut segments. These all contribute to region-dependent nitrergic enteric neuropathy [6,7], that is markedly involved in gut motility disturbances [6,8,9] suffered by diabetic patients worldwide.

The host-microbial interactions have an essential role in starting avalanche-like events, including epithelial barrier functions, intestinal immune cell activation and neuro-immune crosstalk [10]. The neuro-immune interactions have prominent effects on regulating the severity of inflammatory processes [11] in all layers of the gut wall.

Among the key players, tumour necrosis factor alpha (TNFα) has a great impact on the maintenance of intestinal homeostasis under physiological conditions and the dysregulation of TNF signalling may accompany several diseases [12]. The TNF superfamily contains 19 ligands and 29 receptors belonging to transmembrane proteins and characterized by a conserved TNF homology domain at the C-terminal [13]. The two types of TNF proteins, TNFα and TNFβ, have more than 50% of sequence homology [14]. TNFα functions as a cytokine and is expressed mainly by immune cells, macrophages [15], but also by neurons in the central or enteric nervous system [16,17,18]. It can bind to two different receptors (TNFR1 and TNFR2) and regulates several cell functions, such as inflammation, immune response during infection, cell proliferation, differentiation or apoptosis [12,19]. After its release, TNFα can enhance the production of other inflammatory cytokines from other cells; therefore, it is primary featured as a powerful pro-inflammatory cytokine [12].

The TNFα-induced enhancement of oxidative stress as well as the consequent neuroinflammatory processes can promote neurodegeneration via different cellular pathways [20]. Nitric oxide-enhanced TNFα-induced neurotoxicity through a suppression of nuclear factor-κB has been observed in retinal neurons [21]. An increased level of TNFα released from activated microglia and astrocytes has been revealed in the affected areas in different central nervous system neurodegenerative diseases [20,22]. The role of an increased TNFα level associated with caspase activation and elevated inducible nitric oxide synthase expression in spinal cord injuries has also been observed in neuronal cell death [23,24]. Additionally, the blockade of TNFα signalling has been in the focus of therapy in many autoimmune diseases such as rheumatoid arthritis or Crohn’s disease [25].

In type 1 diabetic patients, the serum level of TNFα was increased compared to controls [26]. Additionally, elevated plasma TNFα levels correlating with different metabolic parameters have also been revealed [27]. TNFα directly affects insulin signalling and, thus, has a role in the development of insulin resistance and obesity [28,29,30]. TNFα concentration was enhanced even in tear fluids, which correlated well with the severity of diabetic retinopathy [31]. Increased TNFα levels have been related to the development of diabetic nephropathy involving reactive oxygen species accumulation and related cytotoxicity in kidney cells [32,33,34]), while TNFα inhibition ameliorated the glomerular and tubular injury in diabetic kidney [35]. TNFα is directly involved in the destruction of β-cells in isolated islets [36] and plays a key role in the onset of diabetes in mice through the regulation of dendritic cell maturation and activation of islet-specific pancreatic T-cells [37].

The primary goal of the present study is to evaluate the intestinal region-dependent effects of chronic hyperglycaemia and immediate insulin treatment on TNFα expression in myenteric ganglia of different gut segments. Furthermore, we aim to reveal the possible differences in TNFα expression in different layers of the gut wall.

## 2. Materials and Methods

### 2.1. Animal Model

Adult male Wistar rats (Crl:WI BR; Toxi-Coop Zrt., Balatonfüred, Hungary) weighing 210–260 g, kept on standard laboratory chow (Farmer-Mix Kft., Zsámbék, Hungary) and with free access to drinking water, were used throughout the experiments. The rats were divided randomly into three groups: STZ-induced diabetics (diabetics; *n* = 5), insulin-treated STZ-induced diabetics (insulin-treated diabetics; *n* = 4) and sex- and age-matched controls (*n* = 5). Hyperglycaemia was induced as described previously [6,38,39,40]. The animals were considered diabetic if the non-fasting blood glucose concentration was higher than 18 mM. From this time on, the insulin-treated group of hyperglycaemic rats received a subcutaneous injection of insulin (Humulin M3, Eli Lilly Nederland, Utrecht, The Netherlands) each morning (2 IU) and afternoon (2 IU). Equivalent volumes of saline were given subcutaneously to the diabetic and the control rats. The blood glucose level and weight of each animal were measured weekly. Those diabetic animals which recovered spontaneously, or their blood glucose level decreased under 18 mM during the 10-week experimental period were excluded from the study. In all procedures involving experimental animals, the principles of the National Institutes of Health (Bethesda, MD, USA) guidelines and the EU directive 2010/63/EU for the protection of animals used for scientific purposes were strictly followed, and all the experiments were approved by the National Scientific Ethical Committee on Animal Experimentation (National Competent Authority), with the license number XX/1636/2019.

### 2.2. Tissue Handling

Ten weeks after the onset of hyperglycaemia, the animals were killed by cervical dislocation under chloral hydrate anaesthesia (375 mg/kg i.p.). The gut segments of diabetic, insulin-treated diabetic and control rats were dissected and rinsed in 0.05 M phosphate buffer (PB; pH 7.4). Samples were taken from the duodenum (1 cm distal to the pylorus), the ileum (1 cm proximal to the ileo–caecal junction) and the proximal colon, and processed for fluorescent immunohistochemistry, quantitative electron microscopy and enzyme-linked immunosorbent assays (ELISA). For double-labelling fluorescent immunohistochemistry, samples (2–3 mm) from different gut segments were fixed in 4% paraformaldehyde and embedded in melted paraffin. For post-embedding electron microscopy, small pieces (2–3 mm) of the gut segments were fixed in 2% paraformaldehyde and 2% glutaraldehyde solution and then further fixed for 1 h in 1% OsO_4_. After rinsing in buffer and dehydrating in increasing ethanol concentrations and acetone, they were embedded in Embed812 (Electron Microscopy Sciences, Hatfield, PA, USA). For the ELISA, the 3 cm long gut segments were cut along the mesentery and pinched flat. The layer of mucosa and submucosa containing the submucous plexus as well as the layers of intestinal smooth muscle layers, including the myenteric plexus, were snap-frozen in liquid nitrogen and stored at −80 °C until use.

### 2.3. Fluorescent Immunohistochemistry

For double-labelling immunohistochemistry, paraffin sections (3.5 µm) derived from different gut segments were immunostained with TNFα and HuCD. Briefly, after blocking in tris(hydroxymethyl)aminomethane-buffered saline (TBS) containing 1% bovine serum albumin and 10% normal goat serum, the sections were incubated overnight with anti-TNFα (rabbit polyclonal IgG; ab6671, Figure S1, Abcam, Cambridge, UK; final dilution 1:50) and pan-neuronal anti-HuCD (mouse monoclonal IgG; A-21271, Invitrogen, Waltham, MA, USA; final dilution 1:50) primary antibodies at 4 °C. After washing in TBS with 0.025% Triton X-100, sections were incubated with anti-rabbit Alexa Fluor 488 (Life Technologies Corporation, Molecular Probes, Inc., Waltham, MA, USA; final dilution 1:200) and Cy^TM3^ (Jackson ImmunoResearch Laboratories, Inc., West Grove, PA, USA; final dilution 1:200) secondary antibodies for 1 h at room temperature. Negative controls were performed by omitting the primary antibody when no immunoreactivity was observed (Figure S2). Sections were mounted on slides in Fluoromount^TM^ Aqueous Mounting Medium (Sigma-Aldrich, Budapest, Hungary), observed and photographed with a ZEISS Imager Z.2 fluorescent microscope equipped with an Axiocam 506 mono camera.

### 2.4. Quantitative Post-Embedding Immunohistochemistry

Four embed blocks originated from each intestinal segment and condition were used to prepare ultrathin (70 nm) sections which were mounted on nickel grids and processed for TNFα immunogold labelling. Ultrathin sections (three grids per block) were incubated overnight in anti-TNFα rabbit polyclonal IgG (Abcam, UK; final dilution 1:25) primary antibody, followed by colloidal gold-conjugated anti-rabbit IgG (conjugated to 18 nm gold particles; Jackson ImmunoResearch, USA; final dilution 1:20) secondary antibody for 3 h. The specificity of the immunoreaction was assessed in all cases by omitting the primary antibodies in the labelling protocol and incubating the sections only in the gold-conjugated secondary antibodies. Sections were counterstained with uranyl acetate (Merck, Darmstadt, Germany) and lead citrate (Merck, Germany), and were examined and photographed with a JEOL JEM 1400 transmission electron microscope. The quantitative features and the subcellular distributions of the gold particles labelling TNFα were determined in the myenteric ganglia. Fifty digital photographs of five myenteric ganglia per intestinal segment per condition were conducted at a magnification of 20,000× with the AnaySIS 3.2 program (Soft Imaging System GmbH, Münster, Germany). The intensity of the labelling was expressed as the total number of gold particles per unit area.

### 2.5. Measurement of Tissue TNFα Concentrations

The intestinal tissue samples, including the layer of mucosa and submucosa with the submucous plexus (MUC-SUBMUC-SP) or the intestinal smooth muscle layers with the myenteric plexus in between (MUSCLE-MP), were frozen in liquid nitrogen, crushed into powder in a mortar and homogenized in 500 µL homogenizing buffer (100 µL Protease Inhibitor Cocktail (Sigma-Aldrich, Hungary) in 20 mL 0.05 M PB). Tissue homogenates were centrifuged at 5000 rpm for 20 min at 4 °C. The TNFα level of the intestinal tissue samples was determined by means of quantitative ELISA according to the manufacturer’s instructions (SunRed Biotechnology, Shanghai, China). Optical density was measured at 450 nm (Benchmark Microplate Reader; Bio-Rad, Budapest, Hungary). The tissue TNFα concentrations were expressed as pg/mg protein.

### 2.6. Bradford Protein Micromethod for the Determination of Tissue Protein Content

A commercial protein assay kit was used for the determination of protein content in tissue samples. Bradford reagent was added to each sample. After mixing and following 10 min incubation, the samples were assayed spectrophotometrically at 595 nm. Protein level was expressed as mg protein/mL.

### 2.7. Statistical Analysis

Statistical analysis was performed with one-way analysis of variance (ANOVA) and Newman–Keuls test. All analyses were carried out with GraphPad Prism 6.0 (GraphPad Software, San Diego, CA, USA). A probability of *p* < 0.05 was set as the level of significance. All data were expressed as means ± SEM.

## 3. Results

### 3.1. Disease Characteristics of Experimental Animals

The general characteristics of the control, diabetic and insulin-treated diabetic rats at the end of the 10-week experimental period are shown in Table 1. The diabetic rats were characterized by a significantly increased blood glucose concentration (24.19 ± 0.61 mM) as compared to the age- and sex-matched controls (5.81 ± 0.22 mM). The immediate insulin treatment prevented the extremely high blood glucose level, which was close to the control level during the 10-week experimental period (9.48 ± 0.14 mM). The weight of the animals significantly increased in all groups during the experiment, but the final body weight of diabetic rats was less elevated compared to the control and the insulin-treated diabetic animals.

### 3.2. Presence of TNFα Immunoreactivity in the Intestinal Sections

**Figure 1:**
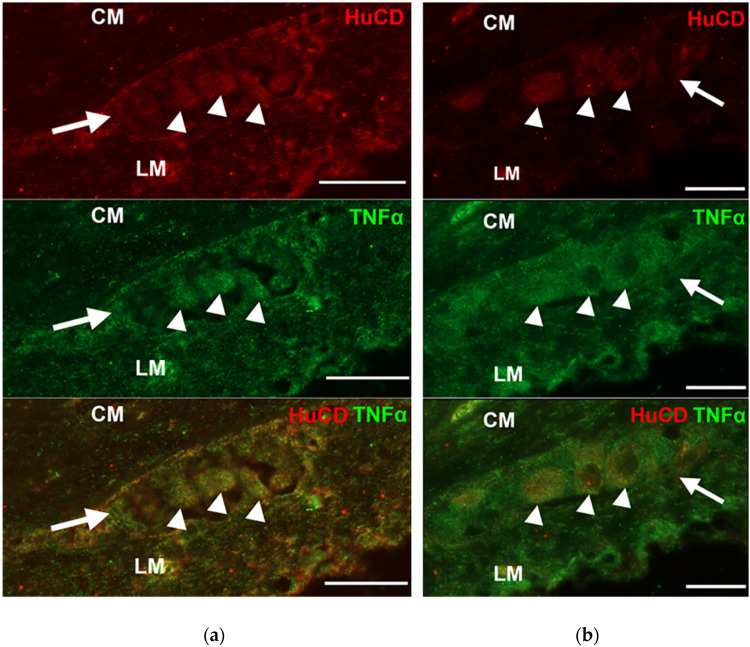
Representative fluorescent micrographs of paraffin sections of myenteric ganglia from the duodenum of a control (**a**) and diabetic (**b**) rat after TNFα-HuCD double-labelling immunohistochemistry. LM—longitudinal smooth muscle layer; CM—circular smooth muscle layer; arrow—myenteric ganglia; arrowheads—myenteric neurons. Scale bar: 20 μm.

Double-labelling fluorescent microscopy revealed TNFα immunoreactivity in myenteric ganglia and their environment (Figure 1). The fluorescent intensity varied in the different intestinal layers: it was clearly visible in the myenteric ganglia, low in the circular smooth muscle and pronouncedly intense in the longitudinal smooth muscle layer (Figure 1).

### 3.3. Subcellular Localization and Quantification of TNFα Expression in Myenteric Ganglia

**Figure 2:**
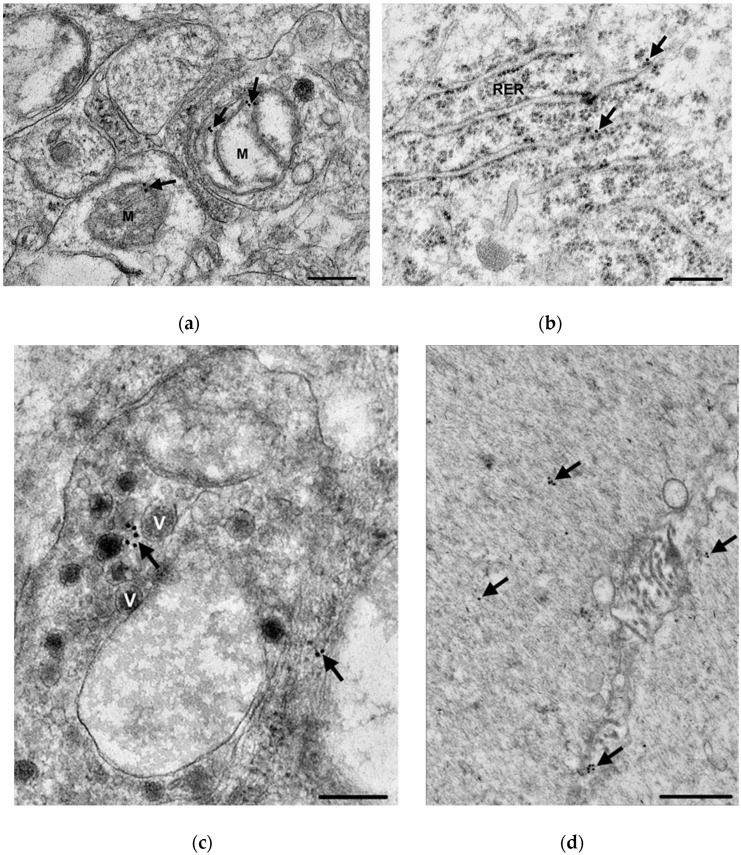
Representative electron micrographs of a portion of the ganglionic neuropil of the myenteric plexus in the duodenum (**a**) and the ileum (**c**) of a diabetic rat, a small detail of a perikaryon from the duodenum (**b**) of a diabetic rat and a part of longitudinal smooth muscle cells of intestinal wall from the colon (**d**) of a control rat after post-embedding immunohistochemistry, using a TNFα-specific antibody. The majority of the 18 nm gold particles labelling TNFα (arrows) were grouped and attached to the plasma membrane or intracellular membranes, and they were observed also in the mitochondria (M) (arrows in (**a**)), in the vicinity of synaptic vesicles (V) (arrows in (**b**)) and at cisternae of the rough endoplasmic reticulum (RER) (arrows in (**c**)). Scale bars: 250 nm.

The presence of TNFα was also confirmed by immunogold electron microscopy in myenteric ganglia (Figure 2). The majority of the 18 nm gold particles labelling TNFα were often located in groups at the plasma membrane or intracellular membranes, such as the mitochondria (Figure 2a) and cisternae of the rough endoplasmic reticulum (Figure 2b) or in the vicinity of synaptic vesicles (Figure 2c).

**Figure 3:**
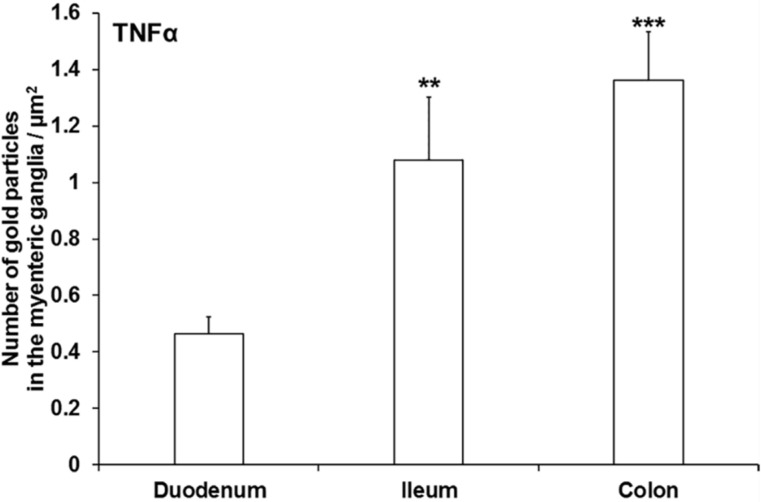
Quantitative evaluation of TNFα-labelling gold particles in the myenteric ganglia from different gut segments of control rats. The numbers of gold particles increased from proximal to distal direction along the gut even in healthy animals. Data were expressed as means ± SEM. ** *p* < 0.01; *** *p* < 0.001 (relative to the control duodenum).

In control animals, the density of TNFα-labelling gold particles was the lowest in the myenteric ganglia of the duodenum (0.46 ± 0.06). In the control ileum, the density of gold labels was more than twice (1.08 ± 0.22; *p* < 0.01) and in the colon it was three times (1.36 ± 0.17; *p* < 0.001) that of the duodenal segment (Figure 3).

**Figure 4:**
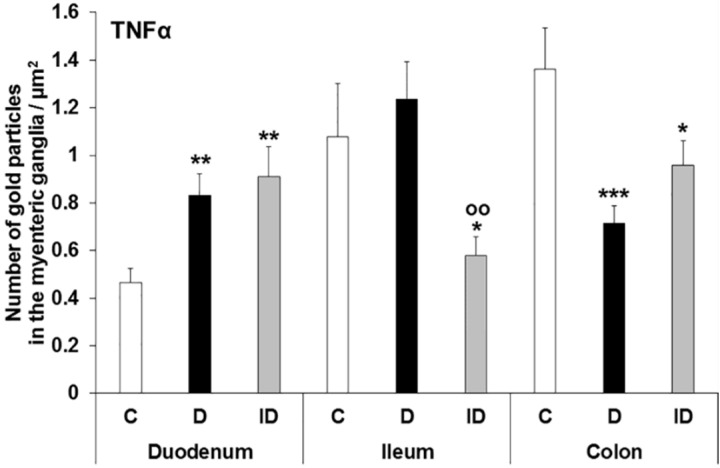
Quantitative evaluation of TNFα-labelling gold particles in the myenteric ganglia from different gut segments of control, diabetic and insulin-treated diabetic rats. The number of TNFα-labelling gold particles increased in the duodenum, decreased in the colon and did not change in the ileum of diabetic rats. The immediate insulin replacement did not prevent the diabetes-related alterations. Data were expressed as means ± SEM. * *p* < 0.05; ** *p* < 0.01; *** *p* < 0.001 (relative to the controls), ^oo^ *p* < 0.01 (between diabetics and insulin-treated diabetics). C–controls; D–diabetics; ID–insulin-treated diabetics.

In the duodenal ganglia of diabetic rats, the number of TNFα-labelling gold particles has nearly doubled relative to controls (0.83 ± 0.09 vs. 0.46 ± 0.06; *p* < 0.01; Figure 4) and it was also elevated in insulin-treated rats (0.91 ± 0.13).

In the ileum of diabetics, the number of TNFα labels did not differ significantly from the control values; however, in insulin-treated rats it was reduced to half compared to the controls (0.58 ± 0.08 vs. 1.08 ± 0.22; *p* < 0.05; Figure 4).

### 3.4. TNFα Levels in Different Intestinal Tissue Layers

**Figure 5:**
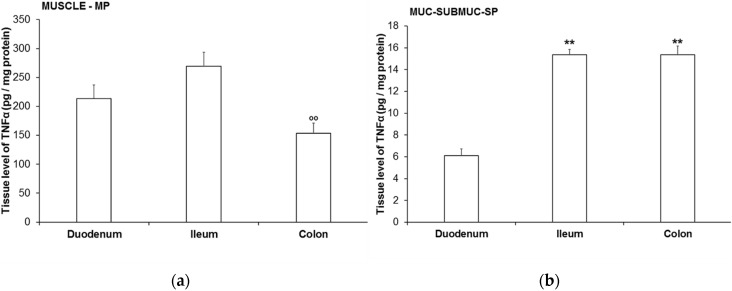
The tissue level of TNFα in the homogenates of intestinal smooth muscle layers, including the myenteric plexus (MUSCLE-MP) (**a**), as well as mucosa and submucosa, including submucous plexus (MUC-SUBMUC-SP) (**b**) from the different gut segments of controls. Data were expressed as means ± SEM. ** *p* < 0.01 (relative to the control duodenum), ^oo^ *p* < 0.01 (between control ileum and colon).

In healthy controls, the tissue level of TNFα varied on an extensive scale between the homogenates of different intestinal layers. While the TNFα protein level was 153.5–269.1 pg/mg in the tissue homogenates of MUSCLE-MP of different gut segments (Figure 5a), it was only 6.1–15.4 pg/mg in homogenates MUC-SUBMUC-SP of different intestinal regions (Figure 5b). The greatest difference was observed in the duodenum, where the TNFα level was 35-times higher in the MUSCLE-MP than in the MUC-SUBMUC-SP. Similarly, considerable differences were revealed in the other segments: the TNFα level was more than 17-times higher in the ileal and 10-times higher in the colonic MUSCLE-MP than in the MUC-SUBMUC-SP (Figure 5).

In control rats, the tissue level of TNFα also varied along the gastrointestinal tract. In the case of MUSCLE-MP samples, it was the lowest in the colon and higher in the ileum (*p* < 0.01) and duodenum (Figure 5a). However, in the case of MUC-SUBMUC-SP samples, the TNFα level was the lowest in the duodenum and it was more than twice as high as in the ileum (*p* < 0.01) and colon (*p* < 0.01) (Figure 5b).

**Figure 6:**
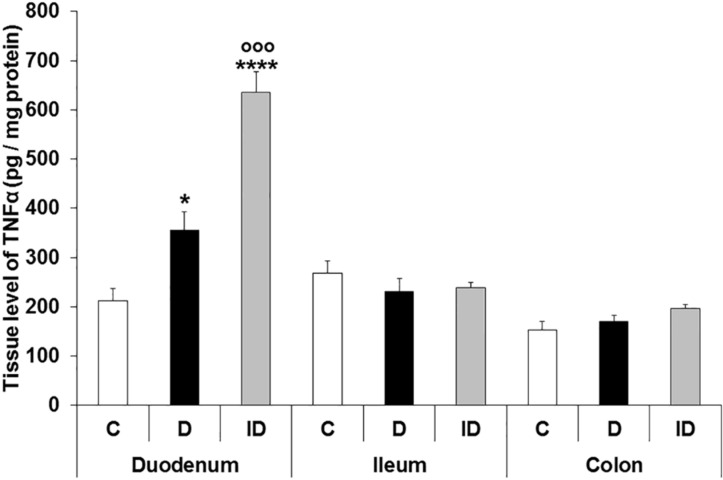
The tissue level of TNFα in the homogenates of intestinal smooth muscle layers, including the myenteric plexus, from the different gut segments of control, diabetic and insulin-treated diabetic rats. The tissue level of TNFα was markedly increased in the duodenum of diabetic animals, which was further enhanced in the insulin-treated diabetics. Data were expressed as means ± SEM. * *p* < 0.05; **** *p* < 0.0001 (relative to the controls), ^ooo^  *p* < 0.001 (between diabetics and insulin-treated diabetics). C—controls; D—diabetics; ID—insulin-treated diabetics.

The effects of hyperglycaemia on TNFα expression were also strictly region-dependent. On the one hand, in MUSCLE-MP homogenates, the tissue level of TNFα increased significantly only in the duodenum of diabetics relative to controls (356.1 ± 36.93 vs. 213.1 ± 23.68; *p* < 0.05); moreover, a further increase was observed in the insulin-treated diabetic group (635.1 ± 41.91; *p* < 0.0001). In contrast, no significant differences were detected in the MUSCLE-MP tissue homogenates of the ileum and colon between control and diabetic groups or even after insulin replacement (Figure 6).

**Figure 7:**
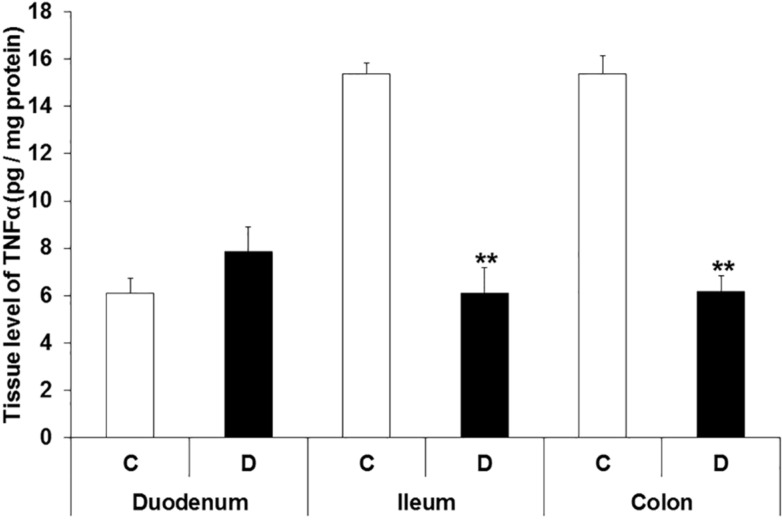
The tissue level of TNFα in the homogenates of mucosa and submucosa, including submucous plexus, from the different gut segments of control and diabetic rats. The level of TNFα was the lowest in the tissue homogenates from the duodenum of controls, while it was more than twice as high in the distal part of the gastrointestinal tract. A statistically significant decrease was observed in TNFα levels in the tissue homogenates of ileum and colon, but not of the duodenum of diabetic rats. Data were expressed as means ± SEM. ** *p* < 0.01 (between controls and diabetics). C—controls; D—diabetics.

On the other hand, in the MUC-SUBMUC-SP samples of diabetic rats, the TNFα level was unchanged in the duodenum, while significantly decreased in the ileum and colon relative to controls (6.1 ± 1.08 vs. 15.37 ± 0.47 in the ileum, *p* < 0.01; 6.18 ± 0.65 vs. 15.36 ± 0.79 in the colon, *p* < 0.01) and (Figure 7 and Figure S3).

## 4. Discussion

In the present study, a distinct gut region-dependent TNFα induction was revealed in type 1 diabetic rats and segment-specific effects of immediate insulin treatment on TNFα expression was also observed. These findings agreed well with the regionality of diabetic myenteric neuropathy [6] and regional damage of enteric neuronal environment in diabetes [3,38,39,40,41]. Moreover, TNFα levels also showed intestinal layer-dependent differences in health and diabetes as confirmed by immunofluorescence, immunogold electron microscopy and ELISA.

In the control state, an increasing tendency of TNFα-labelling gold particle density was observed in myenteric ganglia along the proximo-distal axis of the gut. A three times higher basal density of TNFα gold labels in the colon than in the proximal small intestine meant a great disparity in itself and refers to a more unfavourable baseline environment and prooxidant milieu of the colon [3,42]. Interestingly, in the colonic myenteric ganglia, the number of TNFα gold particles was half of the control value in diabetic rats. Alteration in the endogenous heme oxygenase (HO), expression may have served as a possible explanation of this finding. In the colon, diabetes-related induction of the HO system and an elevated number of HO1-immunoreactive myenteric neurons was demonstrated [39], which may contribute to the decrease in TNFα expression in ganglia of this gut segment. In mixed cultures of adipocytes and macrophages, the induction of HO1 expression resulted in a decrease in TNFα secretion and production of other inflammatory mediators; therefore, attenuating the inflammatory responses [43,44]. Additionally, the silencing of HO1 expression increased TNFα secretion in the same cell culture [43]. Induced HO1 expression also supressed the TNFα-mediated inflammatory processes in human alveolar epithelial cells [45]. In the colon of diabetic rats, we showed markedly decreased TNFα levels also in the tissue homogenates of MUC-SUBMUC-SP samples, which may refer to a similar response in the mucosal–submucosal layers of the large intestine.

In ileal myenteric ganglia, we did not observe any significant alterations in the density of TNFα gold particles between controls and diabetics, despite of the great induction of the HO system here [39]. However, similar to the colon, a robust decrease in the TNFα level was demonstrated in MUC-SUBMUC-SP samples of the diabetic ileum, which may be a result of a mucosal immune response reflecting the enhanced intestinal HO [39] induced by the diabetes-related massive changes in the microbial composition of these distal gut segments [3].

In the myenteric ganglia of duodenum, the density of TNFα-labelling gold particles almost doubled in diabetics relative to controls. Increasing the tissue level of TNFα was also confirmed in MUSCLE-MP homogenates prepared from diabetic duodenum, which may derive mainly from the higher amount of intestinal smooth muscle in the homogenates. The most intense fluorescence labelling was also detected in the longitudinal smooth muscle. However, the TNFα level did not change significantly in duodenal MUC-SUBMUC-SP samples of diabetics. Continuing the evaluation of the possible crosstalk between HO and TNFα expression, it should be noted that according to our earlier finding, the duodenum was the only gut segment, in which the number of HO1- and HO2-immunoreactive neurons did not increase; it remained unchanged in the myenteric ganglia of diabetics [39].

It is known that the expression of different pro-inflammatory cytokines, such as TNFα, is under an extraordinarily complex regulation [46,47], and the host-microbial interactions, are crucial in this process [48,49,50]. In a recent study [4], striking differences were revealed in the mucosa-associated duodenal microbiota of diabetic rats. Diabetic duodenal samples were characterized by a massive invasion of the genus Mycoplasma (14%), while this invasion was not detected in control samples at all. Mycoplasma species are involved in several diseases affecting different organs in patients, and they definitely affect TNFα production. For instance, in children with pneumonia, Mycoplasma infection increases the serum concentration of TNFα that is in correlation with the severity of the disease [51,52,53]. Mycoplasmas markedly enhance TNFα production in cultured astrocytes [54]. Moreover, mycoplasmas differently trigger the innate immune responses of various antigen presenting cells; they activate strong TNF responses in monocytes, induce B-cells, but poorly affect the TNF expression of dendritic cells [55]. Enteric neurons are also capable of producing different cytokines such as TNFα or interleukins in response to lipopolysaccharide [18,56], suggesting the possibility of the enteric nervous system to initiate defence against pathogenic stimuli [57,58].

However, TNFα has a dual role in cell survival/cell death via different signalling pathways depending on its receptors, TNFR1 and TNFR2 [59]. Whereas TNFR1 is mainly involved in inflammation and neurodegenerative processes, TNFR2 contributes to cell regeneration and neuroprotection [17,20,59]. Since in the duodenum of diabetic rats the total number of myenteric neurons remained unchanged [6], we presumed that an increased duodenal TNFα expression may have participated in neuroprotection here. Naturally, further experiments are needed to analyse the TNFR distribution and TNFα signalling in different gut segments for the evaluation of the TNFα effects on enteric neurons.

Immediate insulin treatment did not result in protection against diabetes-related alterations of TNFα expression in any of the gut segments. Moreover, it triggered a great increase in the TNFα level in MUSCLE-MP homogenates relative to both diabetic and control groups. According to literature data, increased amount of TNFα indicates macrophage-induced insulin resistance of the skeletal muscle [60], cerebral insulin resistance in Alzheimer’s disease [61] and it inhibits the effect of insulin on glucose uptake and storage [62,63].

As we mentioned earlier, the tissue level of TNFα showed an intestinal layer-dependent concentration even in physiological conditions. The MUSCLE-MP samples displayed orders of magnitude higher TNFα concentration than MUC-SUBMUC-SP homogenates in controls. A similar tendency was observed in the distal colon of mice, where the myenteric TNFα level was multiple times higher than those of the mucosa [64]. The robust difference in TNFα levels between the different layers of the gut wall supports our previous finding that the myenteric plexus is more vulnerable to oxidative stress than submucous ganglia [5]. Furthermore, it suggests a sensitive regulatory role for TNFα in microbiota neuro-immune crosstalk.

## 5. Conclusions

In conclusion, the present study demonstrated that chronic hyperglycaemia affects the expression of TNFα in a strictly regional and intestinal layer-dependent manner and it contributes to the region-specific damage of myenteric neurons and their environment in rats with type 1 diabetes. TNFα as a potential therapeutic target has long been in the focus of medical research [60,61,62,63]. However, to draw functional conclusions or medical consequences from these findings, further regional investigations involving other elements of TNF signalling pathways are needed.

## Figures and Tables

**Table 1:** Weight and glycaemic characteristics of the three experimental rat groups.

	Body Weight (g) ± SEM		Blood Glucose Concentration (mmol/L) ± SEM	
	Initial	Final	Initial	Final (Average)
Controls (*n* = 5)	224.4 ± 7.13	504 ± 15.65 ^a^	5.96 ± 0.34	5.81 ± 0.22
Diabetics (*n* = 5)	228.4 ± 7.19	373.4 ± 12.51 ^ab^	6.2 ± 0.25	24.19 ± 0.61 ^ab^
Insulin-treated diabetics (*n* = 4)	251.5 ± 4.35	481.5 ± 13.4 ^ac^	6.65 ± 0.18	9.48 ± 0.14 ^abc^

^a^  *p* < 0.0001 initial vs. final, ^b^  *p* < 0.0001 vs. final controls, ^c^  *p* < 0.0001 vs. final diabetics.

## Data Availability

Available upon request.

## Supplementary Materials

The following are available online at www.mdpi.com/2073-4409/10/9/2410/s1, Figure S1: Datasheet of TNFα antibody, Figure S2: Representative fluorescent micrograph of a paraffin section of myenteric ganglia from the ileum of a control rat showing negative control by omitting the primary antibodies against TNFα (green) and HuCD (red). Fluorescent mounting medium containing DAPI was applied for better visualization of the structure. LM-longitudinal smooth muscle layer, CM-circular smooth muscle layer, arrow-myenteric ganglia. Scale: 10 µm, Figure S3: Representative fluorescent micrographs of paraffin sections of mucosal layers originated from the colon of a control (a) and diabetic (b) rat after TNFα fluorescent immunohistochemistry. Scale bar: 100 μm.

## References

[1] Burns A.J., Pachnis V. (2009). Development of the enteric nervous system: Bringing together cells, signals and genes. Neurogastroenterol. Motil..

[2] Brown H., Esterhazy D. (2021). Intestinal immune compartmentalization: Implications of tissue specific determinants in health and disease. Mucosal Immunol..

[3] Wirth R., Bodi N., Maroti G., Bagyanszki M., Talapka P., Fekete E., Bagi Z., Kovacs K.L. (2014). Regionally distinct alterations in the composition of the gut microbiota in rats with streptozotocin-induced diabetes. PLoS ONE.

[4] Wirth R., Bódi N., Szalai Z., Chandrakumar L., Maróti G., Kovács K.L., Bagi Z., Mezei D., Balázs J., Bagyánszki M. (2021). Perturbation of the mucosa-associated anaerobic gut microbiota in streptozotocin-induced diabetic rats. Acta Biol. Szeged..

[5] Bodi N., Bagyanszki M., Preedy V.R. (2020). Diabetic enteric neuropathy: Imbalance between oxidative and antioxidative mechanisms. Diabetes: Oxidative Stress and Dietary Antioxidants.

[6] Izbeki F., Wittman T., Rosztoczy A., Linke N., Bodi N., Fekete E., Bagyanszki M. (2008). Immediate insulin treatment prevents gut motility alterations and loss of nitrergic neurons in the ileum and colon of rats with streptozotocin-induced diabetes. Diabetes Res. Clin. Pract..

[7] Bodi N., Szalai Z., Chandrakumar L., Bagyanszki M. (2017). Region-dependent effects of diabetes and insulin-replacement on neuronal nitric oxide synthase- and heme oxygenase-immunoreactive submucous neurons. World J. Gastroenterol..

[8] Yarandi S.S., Srinivasan S. (2014). Diabetic gastrointestinal motility disorders and the role of enteric nervous system: Current status and future directions. Neurogastroenterol. Motil..

[9] Klinge M.W., Sutter N., Mark E.B., Haase A.M., Borghammer P., Schlageter V., Lund S., Fleischer J., Knudsen K., Drewes A.M. (2021). Gastric Emptying Time and Volume of the Small Intestine as Objective Markers in Patients with Symptoms of Diabetic Enteropathy. J. Neurogastroenterol. Motil..

[10] Caputi V., Popov J., Giron M.C. (2021). Gut Microbiota as a Mediator of Host Neuro-Immune Interactions: Implications in Neuroinflammatory Disorders. Mod. Trends Psychiatry.

[11] Chandrasekharan B., Jeppsson S., Pienkowski S., Belsham D.D., Sitaraman S.V., Merlin D., Kokkotou E., Nusrat A., Tansey M.G., Srinivasan S. (2013). Tumor necrosis factor-neuropeptide Y cross talk regulates inflammation, epithelial barrier functions, and colonic motility. Inflamm. Bowel Dis..

[12] Ruder B., Atreya R., Becker C. (2019). Tumour Necrosis Factor Alpha in Intestinal Homeostasis and Gut Related Diseases. Int. J. Mol. Sci..

[13] Vanamee E.S., Faustman D.L. (2018). Structural principles of tumor necrosis factor superfamily signaling. Sci. Signal..

[14] Aggarwal B.B., Kohr W.J., Hass P.E., Moffat B., Spencer S.A., Henzel W.J., Bringman T.S., Nedwin G.E., Goeddel D.V., Harkins R.N. (1985). Human tumor necrosis factor. Production, purification, and characterization. J. Biol. Chem..

[15] Parameswaran N., Patial S. (2010). Tumor necrosis factor-alpha signaling in macrophages. Crit. Rev. Eukaryot Gene Expr..

[16] Aggarwal B.B., Gupta S.C., Kim J.H. (2012). Historical perspectives on tumor necrosis factor and its superfamily: 25 years later, a golden journey. Blood.

[17] Marchetti L., Klein M., Schlett K., Pfizenmaier K., Eisel U.L. (2004). Tumor necrosis factor (TNF)-mediated neuroprotection against glutamate-induced excitotoxicity is enhanced by N-methyl-D-aspartate receptor activation. Essential role of a TNF receptor 2-mediated phosphatidylinositol 3-kinase-dependent NF-kappa B pathway. J. Biol. Chem..

[18] Coquenlorge S., Duchalais E., Chevalier J., Cossais F., Rolli-Derkinderen M., Neunlist M. (2014). Modulation of lipopolysaccharide-induced neuronal response by activation of the enteric nervous system. J. Neuroinflammation.

[19] Barbara J.A., Van Ostade X., Lopez A. (1996). Tumour necrosis factor-alpha (TNF-alpha): The good, the bad and potentially very effective. Immunol. Cell Biol..

[20] Fischer R., Maier O. (2015). Interrelation of oxidative stress and inflammation in neurodegenerative disease: Role of TNF. Oxid. Med. Cell Longev..

[21] Nakaizumi A., Horie T., Kida T., Kurimoto T., Sugiyama T., Ikeda T., Oku H. (2012). Nitric oxide potentiates TNF-alpha-induced neurotoxicity through suppression of NF-kappaB. Cell Mol. Neurobiol..

[22] Smith J.A., Das A., Ray S.K., Banik N.L. (2012). Role of pro-inflammatory cytokines released from microglia in neurodegenerative diseases. Brain Res. Bull..

[23] Chen K.B., Uchida K., Nakajima H., Yayama T., Hirai T., Watanabe S., Guerrero A.R., Kobayashi S., Ma W.Y., Liu S.Y. (2011). Tumor necrosis factor-alpha antagonist reduces apoptosis of neurons and oligodendroglia in rat spinal cord injury. Spine.

[24] Mir M., Asensio V.J., Tolosa L., Gou-Fabregas M., Soler R.M., Llado J., Olmos G. (2009). Tumor necrosis factor alpha and interferon gamma cooperatively induce oxidative stress and motoneuron death in rat spinal cord embryonic explants. Neuroscience.

[25] Kontermann R.E., Scheurich P., Pfizenmaier K. (2009). Antagonists of TNF action: Clinical experience and new developments. Expert Opin. Drug Discov..

[26] Qiao Y.C., Chen Y.L., Pan Y.H., Tian F., Xu Y., Zhang X.X., Zhao H.L. (2017). The change of serum tumor necrosis factor alpha in patients with type 1 diabetes mellitus: A systematic review and meta-analysis. PLoS ONE.

[27] Lechleitner M., Koch T., Herold M., Dzien A., Hoppichler F. (2000). Tumour necrosis factor-alpha plasma level in patients with type 1 diabetes mellitus and its association with glycaemic control and cardiovascular risk factors. J. Intern. Med..

[28] Alzamil H. (2020). Elevated Serum TNF-alpha Is Related to Obesity in Type 2 Diabetes Mellitus and Is Associated with Glycemic Control and Insulin Resistance. J. Obes..

[29] Swaroop J.J., Rajarajeswari D., Naidu J.N. (2012). Association of TNF-alpha with insulin resistance in type 2 diabetes mellitus. Indian J. Med. Res..

[30] Akash M.S.H., Rehman K., Liaqat A. (2018). Tumor Necrosis Factor-Alpha: Role in Development of Insulin Resistance and Pathogenesis of Type 2 Diabetes Mellitus. J. Cell Biochem..

[31] Costagliola C., Romano V., De Tollis M., Aceto F., dell’Omo R., Romano M.R., Pedicino C., Semeraro F. (2013). TNF-alpha levels in tears: A novel biomarker to assess the degree of diabetic retinopathy. Mediators Inflamm..

[32] Donate-Correa J., Ferri C.M., Sanchez-Quintana F., Perez-Castro A., Gonzalez-Luis A., Martin-Nunez E., Mora-Fernandez C., Navarro-Gonzalez J.F. (2020). Inflammatory Cytokines in Diabetic Kidney Disease: Pathophysiologic and Therapeutic Implications. Front Med..

[33] Purohit S., Sharma A., Zhi W., Bai S., Hopkins D., Steed L., Bode B., Anderson S.W., Reed J.C., Steed R.D. (2018). Proteins of TNF-alpha and IL6 Pathways Are Elevated in Serum of Type-1 Diabetes Patients with Microalbuminuria. Front Immunol..

[34] Navarro J.F., Mora-Fernandez C. (2006). The role of TNF-alpha in diabetic nephropathy: Pathogenic and therapeutic implications. Cytokine Growth Factor Rev..

[35] Cheng D., Liang R., Huang B., Hou J., Yin J., Zhao T., Zhou L., Wu R., Qian Y., Wang F. (2021). Tumor necrosis factor-alpha blockade ameliorates diabetic nephropathy in rats. Clin. Kidney J..

[36] Thomas H.E., Darwiche R., Corbett J.A., Kay T.W. (1999). Evidence that beta cell death in the nonobese diabetic mouse is Fas independent. J. Immunol..

[37] Lee L.F., Xu B., Michie S.A., Beilhack G.F., Warganich T., Turley S., McDevitt H.O. (2005). The role of TNF-alpha in the pathogenesis of type 1 diabetes in the nonobese diabetic mouse: Analysis of dendritic cell maturation. Proc. Natl. Acad. Sci. USA.

[38] Bodi N., Talapka P., Poles M.Z., Hermesz E., Jancso Z., Katarova Z., Izbeki F., Wittmann T., Fekete E., Bagyanszki M. (2012). Gut region-specific diabetic damage to the capillary endothelium adjacent to the myenteric plexus. Microcirculation.

[39] Chandrakumar L., Bagyanszki M., Szalai Z., Mezei D., Bodi N. (2017). Diabetes-Related Induction of the Heme Oxygenase System and Enhanced Colocalization of Heme Oxygenase 1 and 2 with Neuronal Nitric Oxide Synthase in Myenteric Neurons of Different Intestinal Segments. Oxid. Med. Cell Longev..

[40] Bodi N., Mezei D., Chakraborty P., Szalai Z., Barta B.P., Balazs J., Razga Z., Hermesz E., Bagyanszki M. (2021). Diabetes-related intestinal region-specific thickening of ganglionic basement membrane and regionally decreased matrix metalloproteinase 9 expression in myenteric ganglia. World J. Diabetes.

[41] Jancso Z., Bodi N., Borsos B., Fekete E., Hermesz E. (2015). Gut region-specific accumulation of reactive oxygen species leads to regionally distinct activation of antioxidant and apoptotic marker molecules in rats with STZ-induced diabetes. Int. J. Biochem. Cell. Biol..

[42] Sanders L.M., Henderson C.E., Hong M.Y., Barhoumi R., Burghardt R.C., Carroll R.J., Turner N.D., Chapkin R.S., Lupton J.R. (2004). Pro-oxidant environment of the colon compared to the small intestine may contribute to greater cancer susceptibility. Cancer Lett..

[43] Kim Y., Park Y., Namkoong S., Lee J. (2014). Esculetin inhibits the inflammatory response by inducing heme oxygenase-1 in cocultured macrophages and adipocytes. Food Funct..

[44] Namkoong S., Sung J., Yang J., Choi Y., Jeong H.S., Lee J. (2017). Nobiletin Attenuates the Inflammatory Response Through Heme Oxygenase-1 Induction in the Crosstalk Between Adipocytes and Macrophages. J. Med. Food.

[45] Lin C.C., Hsiao L.D., Cho R.L., Yang C.M. (2019). CO-Releasing Molecule-2 Induces Nrf2/ARE-Dependent Heme Oxygenase-1 Expression Suppressing TNF-alpha-Induced Pulmonary Inflammation. J. Clin. Med..

[46] Kalliolias G.D., Ivashkiv L.B. (2016). TNF biology, pathogenic mechanisms and emerging therapeutic strategies. Nat. Rev. Rheumatol..

[47] Varfolomeev E., Vucic D. (2018). Intracellular regulation of TNF activity in health and disease. Cytokine.

[48] Wang C., Li W., Wang H., Ma Y., Zhao X., Zhang X., Yang H., Qian J., Li J. (2019). Saccharomyces boulardii alleviates ulcerative colitis carcinogenesis in mice by reducing TNF-alpha and IL-6 levels and functions and by rebalancing intestinal microbiota. BMC Microbiol..

[49] Wang Y., Yin Y., Chen X., Zhao Y., Wu Y., Li Y., Wang X., Chen H., Xiang C. (2019). Induction of Intestinal Th17 Cells by Flagellins from Segmented Filamentous Bacteria. Front. Immunol..

[50] Locantore P., Del Gatto V., Gelli S., Paragliola R.M., Pontecorvi A. (2020). The Interplay between Immune System and Microbiota in Osteoporosis. Mediat. Inflamm..

[51] Tian F., Han B., Duan M. (2014). Serum tumor necrosis factor-&alpha; interleukin -6 and galctin-3 concentrations in children with Mycoplasma pneumoniae pneumonia. Zhongguo Dang Dai Er Ke Za Zhi.

[52] Wang Y., Zhang Y., Lu W., Wang L. (2018). Serum Tumor Necrosis Factor-alpha and Interferon-gamma Levels in Pediatric Mycoplasma pneumoniae Pneumonia: A Systematic Review and Meta-Analysis. Can. Respir. J..

[53] Li G., Fan L., Wang Y., Huang L., Wang M., Zhu C., Hao C., Ji W., Liang H., Yan Y. (2019). High co-expression of TNF-alpha and CARDS toxin is a good predictor for refractory Mycoplasma pneumoniae pneumonia. Mol. Med..

[54] Brenner T., Yamin A., Abramsky O., Gallily R. (1993). Stimulation of tumor necrosis factor-alpha production by mycoplasmas and inhibition by dexamethasone in cultured astrocytes. Brain Res..

[55] Trueeb B.S., Braun R.O., Auray G., Kuhnert P., Summerfield A. (2020). Differential innate immune responses induced by Mycoplasma hyopneumoniae and Mycoplasma hyorhinis in various types of antigen presenting cells. Vet. Microbiol..

[56] Burgueno J.F., Barba A., Eyre E., Romero C., Neunlist M., Fernandez E. (2016). TLR2 and TLR9 modulate enteric nervous system inflammatory responses to lipopolysaccharide. J. Neuroinflammation.

[57] Yoo B.B., Mazmanian S.K. (2017). The Enteric Network: Interactions between the Immune and Nervous Systems of the Gut. Immunity.

[58] Giuffre M., Moretti R., Campisciano G., da Silveira A.B.M., Monda V.M., Comar M., Di Bella S., Antonello R.M., Luzzati R., Croce L.S. (2020). You Talking to Me? Says the Enteric Nervous System (ENS) to the Microbe. How Intestinal Microbes Interact with the ENS. J. Clin. Med..

[59] Gough P., Myles I.A. (2020). Tumor Necrosis Factor Receptors: Pleiotropic Signaling Complexes and Their Differential Effects. Front. Immunol..

[60] Bu L., Cao X., Zhang Z., Wu H., Guo R., Ma M. (2020). Decreased secretion of tumor necrosis factor-alpha attenuates macrophages-induced insulin resistance in skeletal muscle. Life Sci..

[61] Clark I., Atwood C., Bowen R., Paz-Filho G., Vissel B. (2012). Tumor necrosis factor-induced cerebral insulin resistance in Alzheimer’s disease links numerous treatment rationales. Pharmacol. Rev..

[62] Rask-Madsen C., Dominguez H., Ihlemann N., Hermann T., Kober L., Torp-Pedersen C. (2003). Tumor necrosis factor-alpha inhibits insulin’s stimulating effect on glucose uptake and endothelium-dependent vasodilation in humans. Circulation.

[63] Halse R., Pearson S.L., McCormack J.G., Yeaman S.J., Taylor R. (2001). Effects of tumor necrosis factor-alpha on insulin action in cultured human muscle cells. Diabetes.

[64] Patel B.A., Fidalgo S., Wang C., Parmar L., Mandona K., Panossian A., Flint M.S., Ranson R.N., Saffrey M.J., Yeoman M.S. (2017). The TNF-alpha antagonist etanercept reverses age-related decreases in colonic SERT expression and faecal output in mice. Sci. Rep..

