# Supplementary material for: Intestinal Region-Specific and Layer-Dependent Induction of TNFα in Rats with Streptozotocin-Induced Diabetes and after Insulin Replacement
Source: Cells. 2021 Sep 13;10(9):2410. doi: 10.3390/cells10092410 (PMC8466257; doi:10.3390/cells10092410)
Supplement: Supplementary file 1 [file cells-10-02410-s001.zip › Bódi et al._ Suppl/FigS1.pdf]

abcam®
discover more

Anti-TNF alpha antibody ab6671

22 Abreviews | 32 References | 5 Images

Overview

**Product name**	Anti-TNF alpha antibody
**Description**	Rabbit polyclonal to TNF alpha
**Specificity**	No reactivity is detected against human TNF-b (lymphotoxin). This antibody will recognize the cell-bound precursor of TNFa as a 26,000 protein in immunoblots, particularly in denatured samples. This antibody is also useful for neutralization of human and primate TNFa activity in bioassays. It does not neutralize the biological activity of lymphotoxin.
**Tested applications**	ELISA, IHC-P, IHC-Fr, Neutralising, WB, ICC/IF, IHC-FoFr
**Species reactivity**	**Reacts with:** Mouse, Rat, Guinea pig, Human, Pig, Fish, Cynomolgus Monkey<br>**Predicted to work with:** Dog, Monkey, Non Human Primates
**Immunogen**	Recombinant full length protein (Human).
**Positive control**	Purchase matching WB positive control:<br>**Human TNF alpha full length protein**
**General notes**	Endotoxin content by LAL is <10 pg/ml.

Properties

**Concentration**	1.000 mg/ml
**Form**	Liquid
**Storage instructions**	Shipped at 4°C. Store at +4°C short term (1-2 weeks). Upon delivery aliquot. Store at -20°C or -80°C. Avoid freeze / thaw cycle.
**Storage buffer**	Preservative: None<br>Constituents: 0.15M Sodium chloride, 0.02M Potassium phosphate. pH 7.2
**Purity**	IgG fraction
**Clonality**	Polyclonal
**Isotype**	IgG

Applications

Our Abpromise guarantee covers the use of **ab6671** in the following tested applications.
The application notes include recommended starting dilutions; optimal dilutions/concentrations should be determined by the end user.

Application	Abreviews	Notes
ELISA		1/200 - 1/1000.
IHC-P	★★★★☆	1/100 - 1/200. Perform heat mediated antigen retrieval before commencing with IHC staining protocol.
IHC-Fr	★★★★★	1/100 - 1/200.
Neutralising		1/200.
WB		

Application	Abreviews	Notes
	★★★	1/500 - 1/2000. Can be blocked with Human TNF alpha full length protein (ab140754).<br>Membrane Blocking is recommended with BSA not Milk for this product.<br>Suitable for use as a positive control for Western blot against recombinant TNFalpha produced in E.Coli.
ICC/IF	★★★★★	Use at an assay dependent concentration. PubMed: 19458984 1:100
IHC-FoFr		Use at an assay dependent concentration.

Target

**Function**	Cytokine that binds to TNFRSF1A/TNFR1 and TNFRSF1B/TNFBR. It is mainly secreted by macrophages and can induce cell death of certain tumor cell lines. It is potent pyrogen causing fever by direct action or by stimulation of interleukin-1 secretion and is implicated in the induction of cachexia, Under certain conditions it can stimulate cell proliferation and induce cell differentiation.
**Involvement in disease**	Genetic variations in TNF are a cause of susceptibility psoriatic arthritis (PSORAS) [MIM:607507]. PSORAS is an inflammatory, seronegative arthritis associated with psoriasis. It is a heterogeneous disorder ranging from a mild, non-destructive disease to a severe, progressive, erosive arthropathy. Five types of psoriatic arthritis have been defined: asymmetrical oligoarthritis characterized by primary involvement of the small joints of the fingers or toes; asymmetrical arthritis which involves the joints of the extremities; symmetrical polyarthritis characterized by a rheumatoidlike pattern that can involve hands, wrists, ankles, and feet; arthritis mutilans, which is a rare but deforming and destructive condition; arthritis of the sacroiliac joints and spine (psoriatic spondylitis).
**Sequence similarities**	Belongs to the tumor necrosis factor family.
**Post-translational modifications**	The soluble form derives from the membrane form by proteolytic processing.<br>The membrane form, but not the soluble form, is phosphorylated on serine residues. Dephosphorylation of the membrane form occurs by binding to soluble TNFRSF1A/TNFR1.<br>O-glycosylated; glycans contain galactose, N-acetylgalactosamine and N-acetylneuraminic acid.
**Cellular localization**	Secreted and Cell membrane.

Anti-TNF alpha antibody images

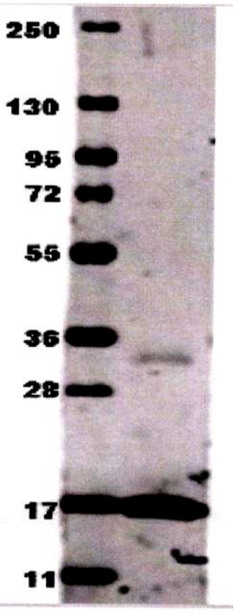

Western blot - Anti-TNF alpha antibody (ab6671)

Anti-TNF alpha antibody (ab6671) at 1/1000 dilution + Recombinant TNF-alpha protein

Secondary
Goat Anti-Rabbit Dylight® 649 Conjugate at 1/20000 dilution

Additional bands at : 16 kDa (possible cleavage fragment). Western Blot analysis, labelling TNF-alpha with ab6671 at 1/1000. Blocking was with 1% BSA in TBS-T incubated for 30 minutes at room temperature. The Cleavage Fragment is related to the soluble isoform 77 - 223.

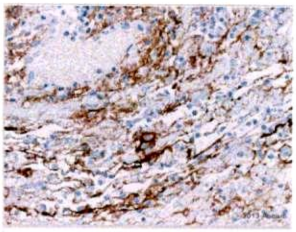

Immunohistochemistry (Formalin/PFA-fixed paraffin-embedded sections) - Anti-TNF alpha antibody (ab6671)

This image is courtesy of an Abreview submitted by Jing Ma

ab6671 staining TNF alpha in Cynomolgus Monkey dendritic cells/macrophages from inlamed skin tissue sections by Immunohistochemistry (IHC-P - paraformaldehyde-fixed, paraffin-embedded sections). Tissue was fixed with formaldehyde and blocked with 10% serum for 20 minutes at room temperature; antigen retrieval was by heat mediation in citrate buffer, pH6.0. Samples were incubated with primary antibody (1/100) for 30 minutes at room temperature. A Biotin-conjugated Goat anti-rabbit IgG polyclonal (1/2000) was used as the secondary antibody.

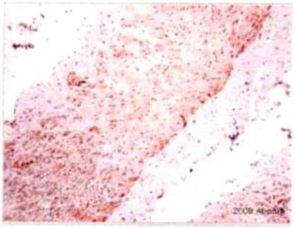

ab6671 staining human artery tissue sections by IHC-P. Sections were fixed in formaldehyde and subjected to heat mediated antigen retrieval in citrate buffer (pH 6.0) prior to blocking with 1.5% serum for 10 minutes. The primary antibody was diluted 1/100 and incubated with the sample for 24 hours at 4°C. An HRP-conjugated goat anti-rabbit antibody was used as the secondary.

Immunohistochemistry (Formalin/PFA-fixed paraffin-embedded sections) - TNF alpha antibody (ab6671)

This image is courtesy of an anonymous Abreview

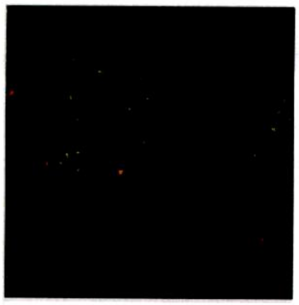

ab6671 staining TNF alpha from human colon by immunohistochemistry (formalin/PFA-fixed paraffin-embedded sections). Cells were formaldehyde fixed prior to blocking in 10% serum for 2 hours at 21°C. The primary antibody was diluted 1/100 and incubated with the sample for 2 hours at 21°C. Alexa fluor® 680 goat polyclonal, diluted 1/5000, was used as the secondary.

Immunohistochemistry (Formalin/PFA-fixed paraffin-embedded sections) - TNF alpha antibody (ab6671)

This image is courtesy of an Abreview submitted by Nicole Schechter

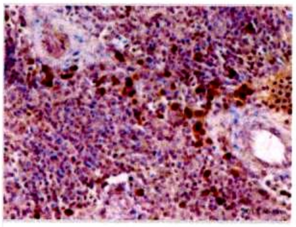

Immunohistochemical analysis of formalin-fixed, paraffin embedded fish tissue, staining TNF alpha with ab6671.

Tissue was fixed with Bouin's fixative; antigen retrieval was by heat mediation in a citrate buffer (pH 6). Samples were incubated with primary antibody (1/600 in diluent) for 2 hours. An HRP-conjugated goat anti-rabbit polyclonal IgG was used as the secondary antibody.

Immunohistochemistry (Formalin/PFA-fixed paraffin-embedded sections) - Anti-TNF alpha antibody (ab6671)

This image is courtesy of an Abreview submitted by Paolo Ronza

Please note: All products are "FOR RESEARCH USE ONLY AND ARE NOT INTENDED FOR DIAGNOSTIC OR THERAPEUTIC USE"

Our Abpromise to you: Quality guaranteed and expert technical support

- Replacement or refund for products not performing as stated on the datasheet
- Valid for 12 months from date of delivery
- Response to your inquiry within 24 hours
- We provide support in Chinese, English, French, German, Japanese and Spanish
- Extensive multi-media technical resources to help you
- We investigate all quality concerns to ensure our products perform to the highest standards

If the product does not perform as described on this datasheet, we will offer a refund or replacement. For full details of the Abpromise, please visit http://www.abcam.com/abpromise or contact our technical team.

Terms and conditions

- Guarantee only valid for products bought direct from Abcam or one of our authorized distributors
