# Supplementary material for: Intestinal Region-Specific and Layer-Dependent Induction of TNFα in Rats with Streptozotocin-Induced Diabetes and after Insulin Replacement
Source: Cells. 2021 Sep 13;10(9):2410. doi: 10.3390/cells10092410 (PMC8466257; doi:10.3390/cells10092410)
Supplement: Supplementary file 1 [file cells-10-02410-s001.zip › Bódi et al._ Suppl/cells-1333415-supplementary.pdf]

Supplementary Materi

See the attached pdf file.

Figure S1. Datasheet of TNFα antibody

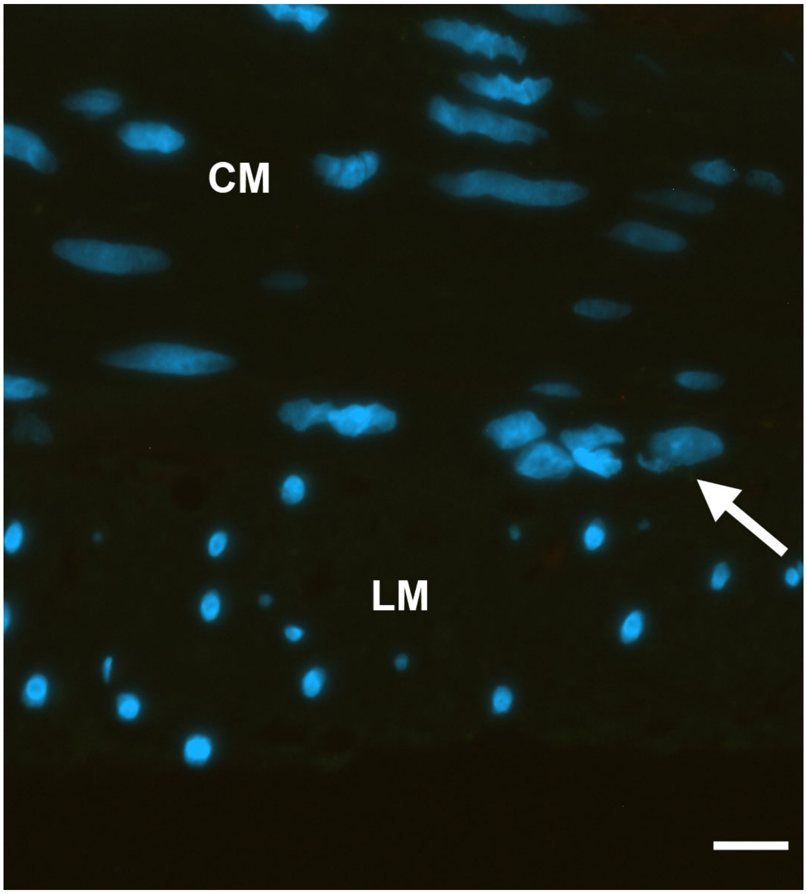

Figure S2. Representative fluorescent micrograph of a paraffin section of myenteric ganglia from the ileum of a control rat showing negative control by omitting the primary antibodies against TNFα (green) and HuCD (red). Fluorescent mounting medium containing DAPI was applied for better visualization of the structure. LM-longitudinal smooth muscle layer, CM-circular smooth muscle layer, arrow-myenteric ganglia. Scale: 10 μm.

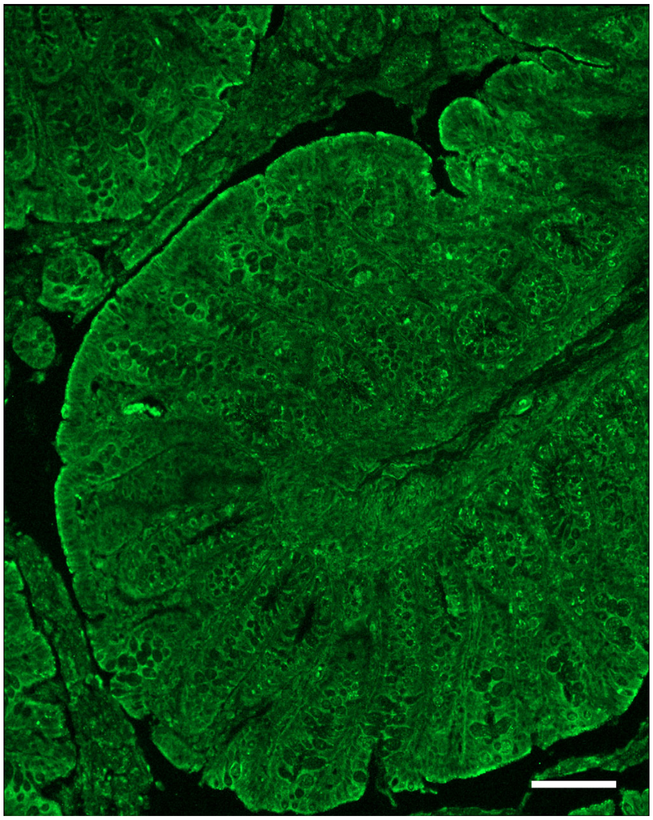

(a)

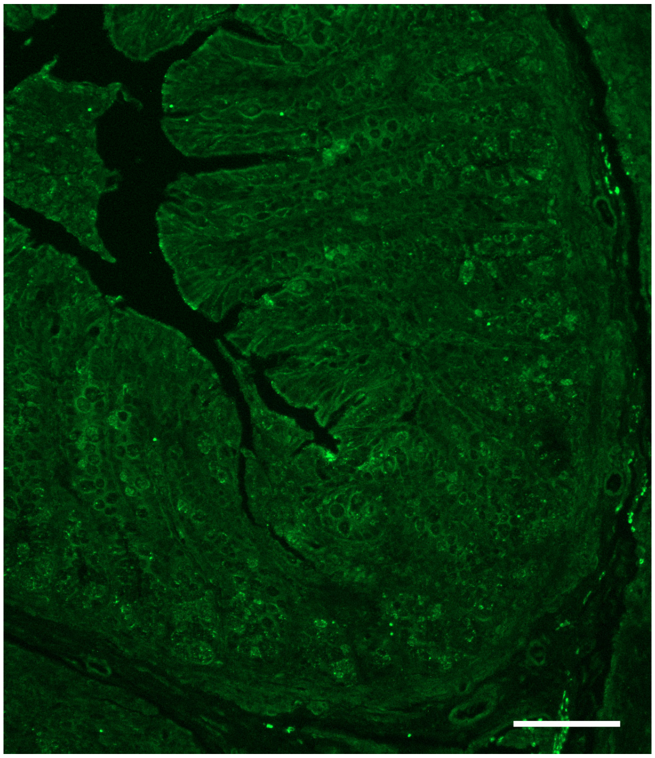

(b)

Figure S3. Representative fluorescent micrographs of paraffin sections of mucosal layers originated from the colon of a control (a) and diabetic (b) rat after TNFα fluorescent immunohistochemistry. Scale bar: 100 μm.
