# Supplementary figures and images for: Intestinal Region-Specific and Layer-Dependent Induction of TNFα in Rats with Streptozotocin-Induced Diabetes and after Insulin Replacement
Source: Cells. 2021 Sep 13;10(9):2410. doi: 10.3390/cells10092410 (PMC8466257; doi:10.3390/cells10092410)

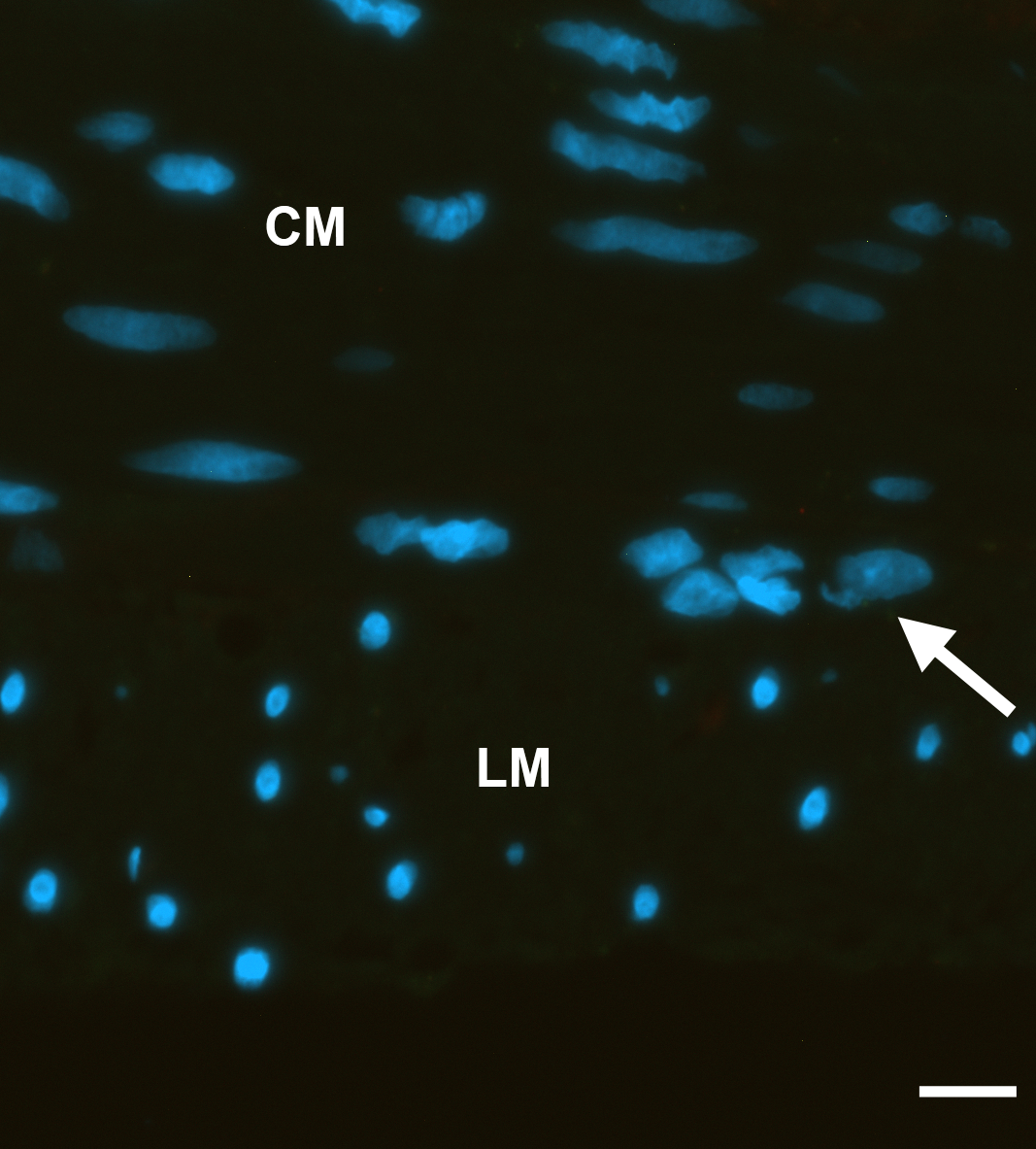

Supplement: Supplementary file 1 [file cells-10-02410-s001.zip › Bódi et al._ Suppl/FigS2.tif]

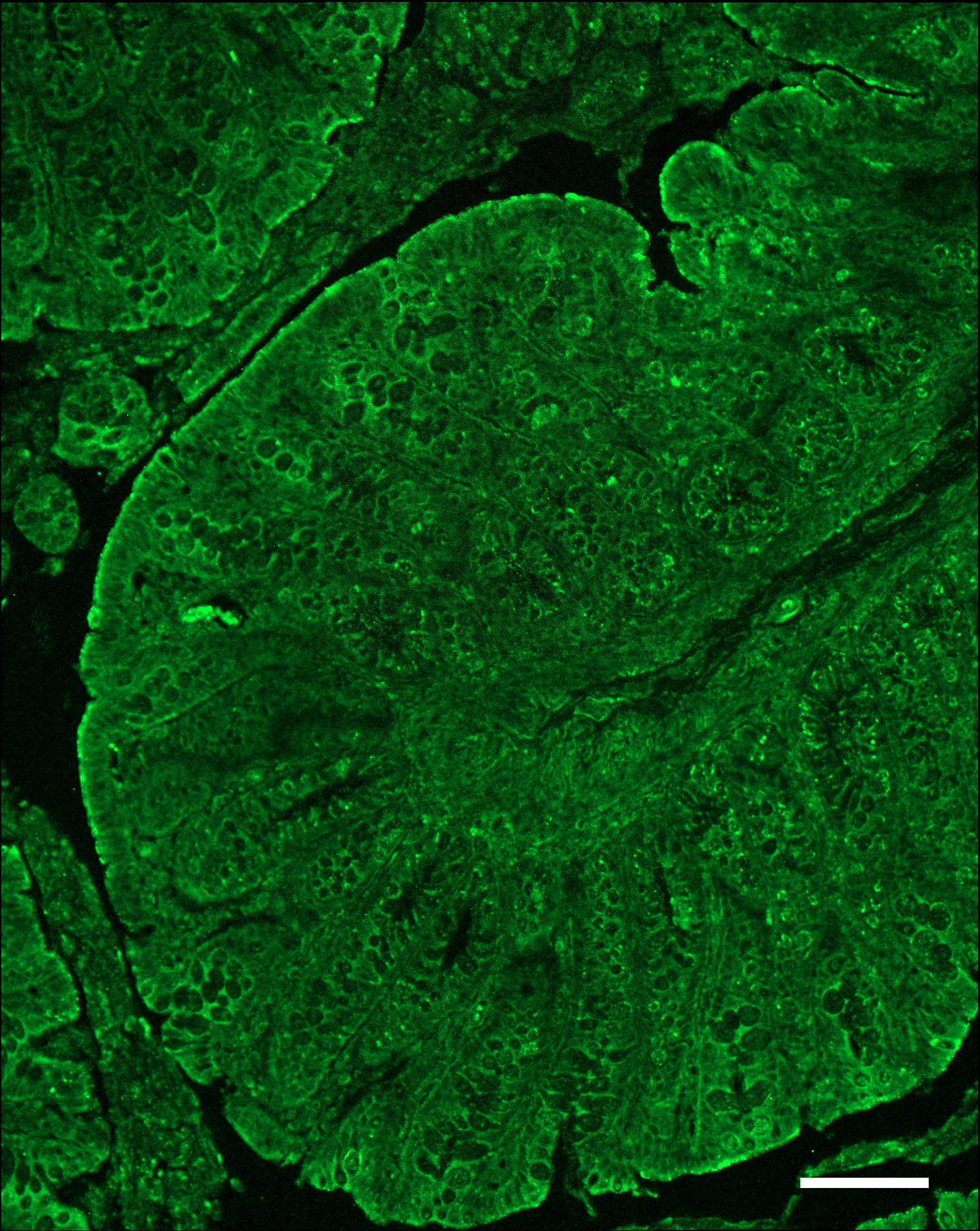

Supplement: Supplementary file 1 [file cells-10-02410-s001.zip › Bódi et al._ Suppl/FigS3a.tif]

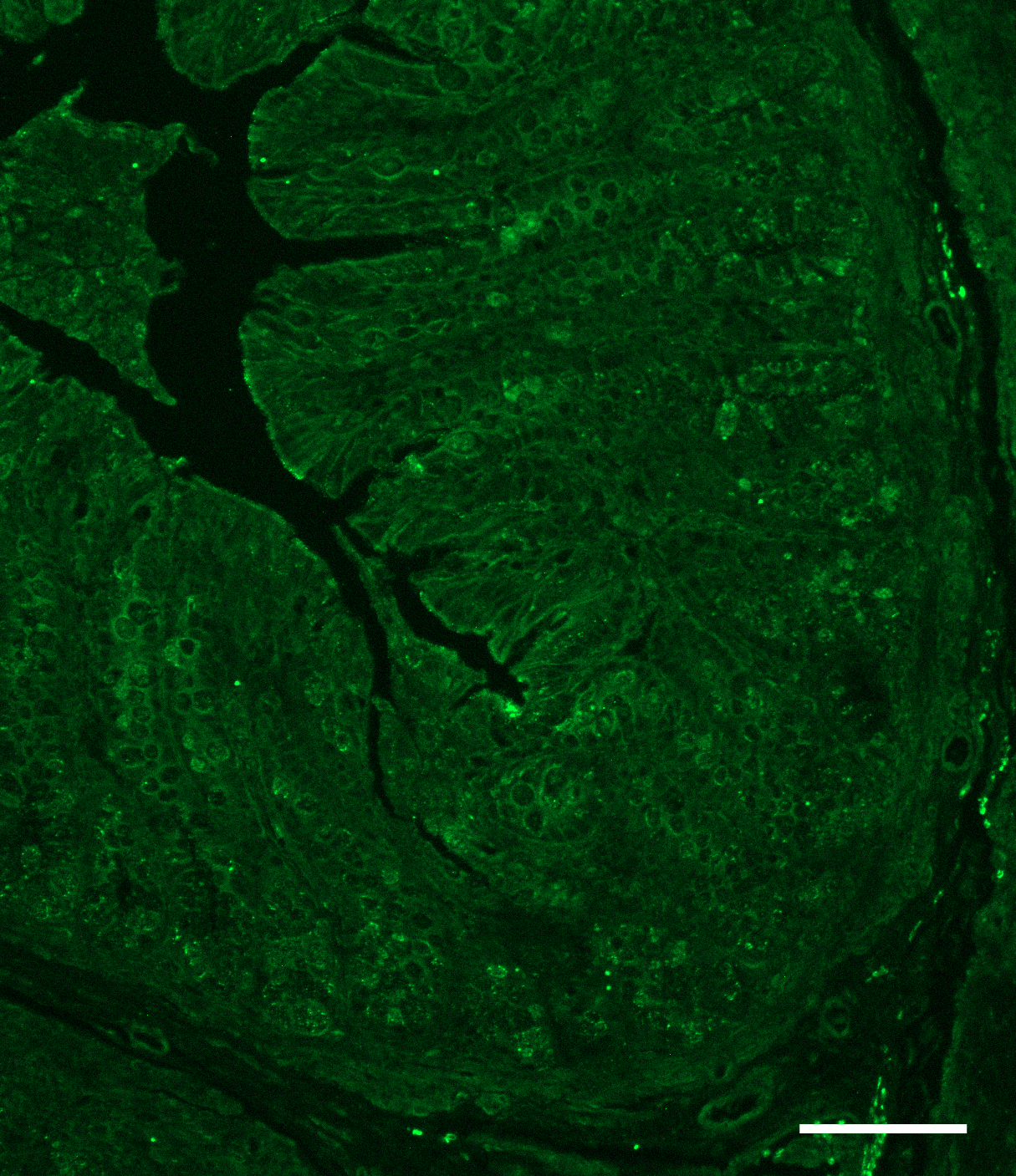

Supplement: Supplementary file 1 [file cells-10-02410-s001.zip › Bódi et al._ Suppl/FigS3b.tif]
